# RETRACTED: Jeong et al. Fermented Sea Tangle (*Laminaria japonica* Aresch) Suppresses RANKL-Induced Osteoclastogenesis by Scavenging ROS in RAW 264.7 Cells. *Foods* 2019, *8*, 290

**DOI:** 10.3390/foods14213656

**Published:** 2025-10-27

**Authors:** Jin-Woo Jeong, Seon Yeong Ji, Hyesook Lee, Su Hyun Hong, Gi-Young Kim, Cheol Park, Bae-Jin Lee, Eui Kyun Park, Jin Won Hyun, You-Jin Jeon, Yung Hyun Choi

**Affiliations:** 1 Nakdonggang National Institute of Biological Resources, Sangju 37242, Republic of Korea; 2 Department of Biochemistry, Dong-eui University College of Korean Medicine, Busan 47227, Republic of Korea; 3 Anti-Aging Research Center, Dong-eui University, Busan 47227, Republic of Korea; 4 Department of Marine Life Sciences, Jeju National University, Jeju 63243, Republic of Koreayoujinj@jejunu.ac.kr (Y.-J.J.); 5 Department of Molecular Biology, College of Natural Sciences, Dong-eui University, Busan 47340, Republic of Korea; 6 Marine Bioprocess Co., Ltd., Busan 46048, Republic of Korea; 7 Department of Oral Pathology and Regenerative Medicine, School of Dentistry, Kyungpook National University, Daegu 41940, Republic of Korea; 8 Department of Biochemistry, School of Medicine, Jeju National University, Jeju 63243, Republic of Korea

The journal retracts the article “Fermented Sea Tangle (*Laminaria japonica* Aresch) Suppresses RANKL-Induced Osteoclastogenesis by Scavenging ROS in RAW 264.7 Cells” [1] cited above.

Following publication, concerns were brought to the attention of the Editorial Office regarding the integrity of a number of images presented in this publication [1].

Adhering to our standard procedure, an investigation was conducted by the Editorial Office and Editorial Board that identified indications of inappropriate editing and partial duplication between figures presented in this article [1] and earlier publications [2,3,4], presenting different experimental conditions. While the authors collaborated within this process, raw material meeting the journal’s Original Images Requirements could not be provided for Editorial Board Evaluation. Consequently, the Editorial Board has lost confidence in the reliability of the findings and has decided to retract this publication, as per MDPI’s retraction policy (https://www.mdpi.com/ethics#_bookmark30, accessed on 6 August 2025).

This retraction was approved by the Editor-in-Chief of the journal *Foods*.

The authors did not agree to this retraction.
